# Repression of class I transcription by cadmium is mediated by the protein phosphatase 2A

**DOI:** 10.1093/nar/gkt335

**Published:** 2013-05-02

**Authors:** Lei Zhou, Gwenaëlle Le Roux, Cécile Ducrot, Stéphane Chédin, Jean Labarre, Michel Riva, Christophe Carles

**Affiliations:** ^1^CEA, iBiTecS, F-91191 Gif-sur-Yvette cedex, France, ^2^CNRS, FRE 3377, F-91191 Gif-sur-Yvette, France and ^3^Université Paris-Sud, FRE 3377, F-91191 Gif-sur-Yvette, France

## Abstract

Toxic metals are part of our environment, and undue exposure to them leads to a variety of pathologies. In response, most organisms adapt their metabolism and have evolved systems to limit this toxicity and to acquire tolerance. Ribosome biosynthesis being central for protein synthesis, we analyzed in yeast the effects of a moderate concentration of cadmium (Cd^2+^) on Pol I transcription that represents >60% of the transcriptional activity of the cells. We show that Cd^2+^ rapidly and drastically shuts down the expression of the 35S rRNA. Repression does not result from a poisoning of any of the components of the class I transcriptional machinery by Cd^2+^, but rather involves a protein phosphatase 2A (PP2A)-dependent cellular signaling pathway that targets the formation/dissociation of the Pol I–Rrn3 complex. We also show that Pol I transcription is repressed by other toxic metals, such as Ag^+^ and Hg^2+^, which likewise perturb the Pol I–Rrn3 complex, but through PP2A-independent mechanisms. Taken together, our results point to a central role for the Pol I–Rrn3 complex as molecular switch for regulating Pol I transcription in response to toxic metals.

## INTRODUCTION

All organisms have to deal with metals that are both essential for their viability, as a large number of proteins require metals for their catalytic activity and/or for maintaining their structure (1), and harmful or toxic depending on their concentration. During evolution, cells have developed a variety of mechanisms to evade toxicity and to acquire tolerance to limit the damaging effects of toxic metals. Indeed, exposure to supraphysiological concentrations of metals leads to organ damage, birth defects, cancer, central nervous system disorders and psychological disorders (2).

Cadmium (Cd^2+^) is a toxic mutagenic metal that was shown to induce apoptosis (3–5) and to cause both oxidative and endoplasmic reticulum stresses (6,7). However, the detailed molecular mechanisms linking exposure to Cd^2+^ to this variety of biological effects have not been unraveled. Cadmium may lead to enzyme inhibition or loss of protein function, via interaction with thiol groups of cysteine residues, and displacement of Zn^2+^ or Ca^2+^ from structural or active sites (8–10). In this respect, the mutagenic character of Cd^2+^ could be related to the inhibition of enzymes of the DNA repair system (11,12), and the Cd^2+^-dependent oxidative stress could be explained by the inhibition of enzymes involved in the control of antioxidant levels or in the metabolism of intracellular iron, leading to high reactive oxygen species (ROS) levels, despite the redox-inactive character of Cd^2+^ (13).

Genome-wide analyses in yeast have shown that Cd^2+^ induces the expression of genes of both the sulfate assimilation and the glutathione biosynthesis pathways (6,14–17). In addition, cells readapt to the high requirement for glutathione by globally modifying their proteome to reduce the production of abundant sulfur-rich proteins (16). The expression of genes that are involved in ribosome biogenesis is also affected by metal exposure (6), allowing resources devoted to ribosome biogenesis to be redirected toward the defense against metal toxicity (18). Indeed, ribosome biogenesis, which monopolizes up to 60% of the transcriptional activity, involves the activities of the three forms of nuclear RNA polymerase (Pol): Pol I and Pol III, respectively, synthesizing the precursors of the large ribosomal RNAs (35S rRNA in yeast) and the 5S rRNA, and Pol II, transcribing the ribosomal protein genes.

Because Pol I activity (i) represents the major transcriptional activity of the cell, (ii) is the key determinant for the level of all ribosomal components (19) and (iii) is tightly coupled to environmental conditions (20), we investigated the response of the class I transcriptional machinery to Cd^2+^ treatment at a moderate metal concentration that does not induce cell lethality. We show here that Cd^2+^ rapidly and drastically shuts down the synthesis of the 35S rRNA by inhibiting the recruitment of Pol I to the ribosomal DNA (rDNA) promoter. We demonstrate that this metal does not directly interfere with any of the components of the class I transcriptional machinery, but rather activates a signal transduction pathway that requires protein phosphatase 2A (PP2A). We also show that the PP2A requirement is not general to metal-dependent repression of Pol I transcription, even though all of the metals tested target the formation/dissociation of the Pol I–Rrn3 complex. Taken together, our results highlight the central role of the Pol I–Rrn3 complex as a molecular switch for regulating class I transcription in response to toxic metals. Interestingly, these observations are the first demonstration that, in yeast, stability of the Pol I–Rrn3 complex is a key element for both the growth-independent and the growth-dependent repression mechanisms of Pol I transcription.

## MATERIALS AND METHODS

### Strains, plasmids and media

Cells were grown in YPD medium at 30°C except when indicated. Strains: YPH500α (*MATα ade2-101 his3-Δ200 leu2-Δ1 lys2-801 trp1-Δ63 ura3-52*) (21); CARA (*MATα ade2-101 his3-Δ200 leu2-Δ1 lys2-801 trp1-Δ63 ura3-52 Δrrn3 ::his5^+^ Δrpa43 ::kan^r^* pGEN-RRN3-RPA43) (19); W303 (*ade2-1 ura3-1 his3-11 trp1-1 leu2-3,112 can1-100 ssd1-d2*) (22) and its isogenic strain DEY217 (*MATa pph22-172::URA3 pph21Δ1::HIS3 pph3Δ1::LYS2 lys2-952*) (22); BY472-Rrn3-HA (*MATα his3Δ 1 leu2Δ 0 lys2Δ 0 ura3Δ 0*, pFA6a-RRN3-3HA-kanMX6) and its isogenic strain BY472 (*MATα his3Δ 1 leu2Δ 0 lys2Δ 0 ura3Δ 0*).

### RNA extraction and analyses

For total RNA extraction, 2.10^8^ cells from exponential phase culture (OD_600_ = 1.0) were recovered by centrifugation, resuspended in 0.5 ml of AE buffer (50 mM sodium acetate buffer, pH 5.3, 10 mM EDTA) with 1% sodium dodecyl sulfate, and mixed with an equal volume of acid-buffered phenol (equilibrated in AE buffer). Cells were disrupted by heating at 65°C for 8 min at 1300 rpm on an Eppendorf Thermomixer and frozen at −80°C. After thawing at room temperature, samples were centrifuged (14 000 *g*, 10 min, room temperature) and, 0.45 ml of the aqueous phase was recovered. An equal volume of phenol-dichloromethane-isoamyl alcohol (25:24:1) was added; then samples were vigorously vortexed at room temperature before being centrifuged (14 000 *g*, 4 min, room temperature). The aqueous phase was recovered, and nucleic acids were precipitated by addition of 2.5 volumes of ethanol and 0.1 volume of 3 M sodium acetate, pH 5.3. The pellet was rinsed with 80% ethanol and dissolved in 40 µl of RNase-free water.

For primer extension, 3 µg of total RNAs extracted from 5.10^6^ cells in exponential phase were analyzed at each point of the time-course analysis, and experiments were performed as described in (23) using the following oligonucleotides as primers:

35S rRNA—5′-TCA CGG AAT GGT ACG TTT GA-3′OH

25S rRNA—5′-TGT TCG CTA TCG GTC TCT C-3′OH

Extension products were separated by electrophoresis on an 8% acrylamide gel containing 7 M urea in TBE. The gel was analyzed with a PhosphorImager (Molecular Dynamics).

### Chromatin immunoprecipitation

Chromatin immunoprecipitations (ChIP) were performed essentially as described (24). At each time point, 50 ml of yeast cultures were harvested in mid-exponential phase (OD_600_ = 1.0) and fixed with 1% formaldehyde for 10 min at room temperature. Glycine was added to a final concentration of 0.4 M, and incubation continued for 5 min. Cells were collected by centrifugation, washed once with cold 20 mM Tris–HCl, pH 8.0, and once with cold FA-lysis buffer (50 mM Hepes-KOH, pH 7.5, 150 mM NaCl, 1 mM EDTA, 1% Triton X-100, 0.1% sodium deoxycholate, 0.1% sodium dodecyl sulfate (SDS), 1 mM phenylmethylsulfonyl fluoride (PMSF), and resuspended in 500 µl of cold FA-lysis buffer. A volume of 750 µl of glass beads (425–600 µm Glass Beads, Sigma) was added, and cells were disrupted by vortexing for 15 min at 4°C. The lysate was diluted into 1.4 ml of cold FA-lysis buffer, and the glass beads were discarded as described (25). The cross-linked chromatin was pelleted by centrifugation (12 000 *g*, 20 min, 4°C), washed with 1.6 ml of cold FA-lysis buffer for 1 h at 4°C, resuspended in 1.6 ml FA-lysis buffer and sonicated to yield an average DNA fragment size of 400 bp (range 100–1000 bp). Finally, the samples were completed with 0.4 ml of cold FA-lysis buffer and clarified by centrifugation (12 000 *g*, 30 min, 4°C).

Chromatin extract (500 µl) was incubated with 5 µl of polyclonal anti-A190 antibodies (26) coupled to sheep anti-rabbit IgG Dynabeads (Dynal). After 1 h on a rotating wheel at room temperature, beads were washed six times: once with 1.4 ml of FA-lysis buffer, three times with 1.4 ml of FA-lysis buffer with a final concentration of 500 mM NaCl, once with 1.4 ml of 10 mM Tris–HCl (pH 8.0), 250 mM LiCl, 1 mM EDTA, 0.5% IGEPAL CA630 (NP-40), 0.5% sodium deoxycholate, and once with 1.4 ml of TE (10 mM Tris–HCl, pH 8.0, 1 mM EDTA). Immunoprecipitated material was eluted from the beads by heating for 20 min at 65°C in 125 µl of 25 mM Tris–HCl, pH 7.5, 5 mM EDTA, 0.5% SDS. After recovering, the cross-link was reversed by incubating the samples with 1 mg.ml^−^^1^ of Pronase (Roche) for 1 h at 37°C and then overnight at 65°C. Samples were treated with 25 µg.ml^−^^1^ of RNase (Eurogentec) for 1 h at 37°C; then DNA was purified using a Qiaquick spin column (Qiagen).

Immunoprecipitated and total DNA samples were quantified in triplicate by real-time polymerase chain reaction (PCR) using the MESA Green qPCR™ Mastermix Plus for SYBR® Assay (Eurogentec) and the 7300 Real-Time PCR System (Applied Biosystems). Sequences of the oligonucleotide primers are available on request. The relative immunoprecipitation (IP) value for a given locus is expressed as a percentage of the occupancy without Cd^2+^ and was calculated as the ratio between the IP signal and the respective total DNA signal, to correct for variation between different samples and primer pairs.

### Co-immunoprecipitation

Cells (2 L) in mid-exponential phase culture (OD_600_ = 0.8) were recovered by centrifugation, suspended in 0.5 ml of extraction buffer [150 mM Hepes, pH 7.8, 60 mM MgCl_2_, 60% glycerol, 3 mM dithiothreitol (DTT)] per gram of cells. The concentration of ammonium sulfate was adjusted at 450 mM, and a cocktail of protease inhibitors (Complete, Roche) was added before disruption of the cells in an Eaton press. Cell extract was centrifuged at 40 000 rpm for 90 min at 4°C in a 50.2 Ti rotor (Beckman). Immunoprecipitation of Rrn3-3HA was performed on 8.2 mg of protein extracts with 12CA5 anti-HA monoclonal antibodies coupled to anti-mouse IgG coated magnetic beads (50 µl, Dynal, Invitrogen) overnight at 4°C with gentle agitation. Beads were washed four times in 500 µl of 30 mM HEPES, pH 7.8, 200 mM potassium acetate, 0.2 mM EDTA, 10 mM MgCl_2_, 0.05% Tween 20, 1 mM DTT, 10% glycerol. Immunoprecipitated proteins were eluted by boiling the beads in Laemmli buffer (27) and analyzed by western blotting. Membranes were probed with mouse 12CA5 anti-HA monoclonal antibodies and rabbit anti-Pol I polyclonal antibodies (26) for the detection of Rrn3 and Pol I, respectively. Immunocomplexes were detected with secondary antibodies conjugated to infrared dyes, scanned and analyzed with the Odyssey imaging system and its associated software (Li-Cor).

### *In vitro* transcription assay

Specific *in vitro* assays using partially purified extracts (PA600) were performed as in (28) with 40 ng of YepSIRT template. Add-back experiments were performed by addition of purified Pol I CARA (0.09 nmoles) (19), ∼0.09 nmoles of rCF (insect cells infected with recombinant Baculovirus), rTBP (*Escherichia coli*) or rUAF (insect cells infected with recombinant Baculovirus) to PA600 extract prepared from WT Cd^2+^-treated cells (50 µM, 30 min).

## RESULTS

Regulation of yeast genome transcription by Pol II in response to Cd^2+^ exposure has been thoroughly studied by high-throughput analyses (6,16), which highlighted a metabolic adaptation of cells, in particular to favor the synthesis of GSH, a key player in metal detoxification (16). However, little is known about the Pol I and Pol III transcriptional responses to this toxic metal. Because the growth rate of yeast cells was significantly reduced after 60 min of exposure to moderate Cd^2+^ concentrations (typically 30 μM, data not shown), and because cellular growth rate and level of 35S rRNA synthesis are highly correlated, we sought to analyze the *in vivo* effect of Cd^2+^ treatment on Pol I transcription.

We first determined the cellular mortality of the yeast *Saccharomyces cerevisiae* induced by different Cd^2+^ concentrations, seeking to determine experimental conditions for which the cell lethality would be minimal to be able to assign potential effects of Cd^2+^ on Pol I transcription as being direct consequences of this metal on rRNA synthesis, rather than indirect effects driven by massive cellular death. We observed that exposure of wild-type (WT) yeast cells to 50 μM or 100 μM of Cd^2+^ led to a modest cellular mortality for short treatment times [95 ± 3% and 90 ± 4% viability for 30 and 60 min of treatment, respectively, with 50 μM Cd^2+^, and 87 ± 4% and 82 ± 5% viability for 30 and 60 min of treatment, respectively, with 100 μM Cd^2+^ (Supplementary Figure S1)]. We therefore performed all experiments in the presence of 50 μM Cd^2+^.

### Cd^2+^ treatment affects Pol I transcription initiation

**Figure 1.**
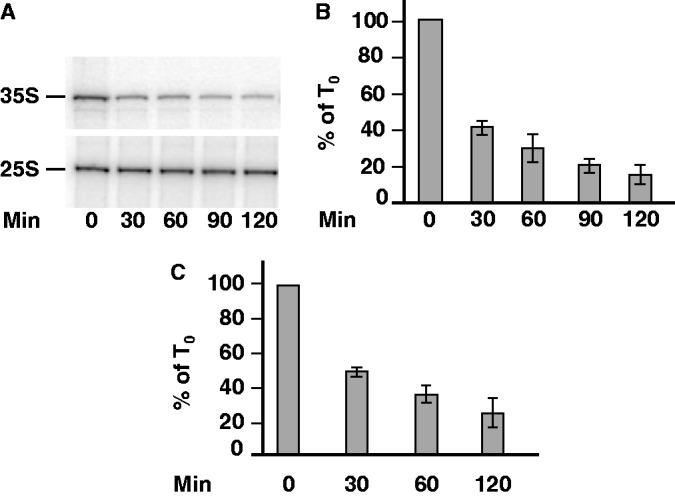
Yeast cells downregulate Pol I transcription in response to Cd^2+^. (**A**) WT cells were grown in YPD medium to mid-exponential phase, and Cd^2+^ (50 µM) was added (*t* = 0 min). At the indicated times, the same number of cells was harvested, total RNAs were extracted and the amount of 35S rRNA was determined by primer extension analysis. (**B**) Quantification of 35S rRNA precursor in four independent primer extension experiments similar to that shown in panel A. (**C**) ChIP analysis of the occupancy of the rDNA unit by Pol I. Cells were grown in YPD medium to mid-exponential phase and Cd^2+^ (50 µM) was added (*t* = 0). At the indicated times, chromatin extracts were prepared from the same number of cells. ChIP experiments were performed using anti-A190 antibodies, and quantification was performed by real-time PCR using four pairs of primers hybridizing at the promoter and in three regions along the coding sequence of the rDNA (see ‘Materials and Methods’ section). The occupancy of rDNA by Pol I being nearly identical for each of these four regions, results were averaged. The occupancy of the rDNA unit by Pol I after Cd^2+^ treatment is represented as percentage of the occupancy seen in the absence of Cd^2+^. Four independent experiments were performed.

To analyze the *in vivo* effect of Cd^2+^ treatment on Pol I transcription, we first determined the level of the precursor of the large ribosomal RNAs (35S rRNA) by primer extension during a time-course analysis. As shown in Figure 1A and B, in the presence of 50 µM Cd^2+^, the *in vivo* level of 35S rRNA decreased dramatically, and reached, after 2 h of treatment, 15% of the 35S rRNA level measured in untreated control cells.

Because the level of 35S RNA monitored by primer extension analysis does not directly reflect Pol I activity, but rather an equilibrium between rDNA transcription, pre-rRNA stability and pre-rRNA processing, we next investigated the effect of Cd^2+^ treatment on the occupancy of the 35S rDNA promoter by Pol I using ChIP. As shown in Figure 1C, the presence of 50 µM Cd^2+^ strongly reduced occupancy of the rDNA promoter by Pol I. After 2 h of Cd^2+^ treatment, rDNA units were occupied by ∼80% fewer Pol I molecules than in the absence of Cd^2+^. Interestingly, a robust correlation was observed between the decreasing occupancy of Pol I on the rDNA unit, and the level of 35S rRNA measured *in vivo* (compare Figure 1B and C), indicating that the drastic drop of 35S rRNA level induced by Cd^2+^ is a direct consequence of the inhibition of Pol I recruitment onto the rDNA promoter. To confirm this hypothesis, we next investigated by ChIP the occupancy by Pol I of the transcribed region of the rDNA, using probes specifically targeting three regions (E1, E2 and E3, Supplementary Figure S2A) distributed all over the coding sequence of the gene. Variations of Pol I occupancy during time-course analyses were similar for the E1, E2 and E3 regions of the rDNA, and identical to those observed on the rDNA promoter (Supplementary Figure S2B), suggesting that steps of the Pol I transcription cycle downstream of initiation (i.e. promoter escape, elongation and termination) were not significantly affected during Cd^2+^ treatment.

### Cd^2+^-dependent repression of Pol I transcription is mediated through PP2A

We first wondered whether the Cd^2+^-dependent repression of the Pol I transcriptional machinery occurred through a direct mechanism, i.e. by affecting the activity of the Pol I or of a class I general transcription factor (GTF). Indeed, Cd^2+^ is known to be highly reactive with sulfydryl groups, and/or may be exchanged with Zn^2+^ atoms present in proteins (8–10), possibly leading to functional defects of the modified proteins. Because numerous components of the class I transcriptional machinery (including Pol I itself) can be poisoned by both mechanisms, we tested the effect of Cd^2+^, in a promoter-dependent *in vitro* transcription assay. A partially purified yeast extract fraction (PA600) containing all the components required for the specific *in vitro* transcription of a rDNA unit was pre-incubated for 3 h at 30°C with different concentrations of Cd^2+^ together with a plasmid harboring a mini-rDNA unit (28). RNA synthesis was started by addition of nucleotides triphosphate (NTPs). Note that, in the absence of Cd^2+^, the efficiency of RNA synthesis was affected by pre-incubation times >3 h at 30°C. We observed that the presence of Cd^2+^ in the reaction mixture did not affect the specific transcription of the template by Pol I (Supplementary Figure S3). Even though this result was obtained *in vitro*, we postulated that the Cd^2+^-dependent repression of the *in vivo* 35S rRNA synthesis was not due to a direct effect of this metal on the class I transcriptional machinery.

Next, we asked whether Cd^2+^-dependent Pol I transcription repression could be mediated by a particular signaling pathway because, in addition to the cell damage it causes, exposure to metals is known to activate a variety of intracellular signal transduction pathways [reviewed in (29)]. In yeast, pathways responsible for the cellular response to metal exposure have been tentatively decrypted by analyzing the transcriptome and the deletome profiles of yeast cells exposed to transition metals (6). Genomic profiles defined by these high-throughput analyses were integrated to identify cellular pathways required for cell survival through regulation of gene expression under toxic conditions. In particular, endoplasmic reticulum (ER) stress response, Hog1, Pkc1, Snf1, cAMP-dependent PK and PP2A signaling pathways have been pointed out as being involved in the response of yeast cells to Cd^2+^ treatment (6), and we wondered whether one or several of these pathways were required for the Cd^2+^-dependent repression of Pol I transcription. To address this issue, we analyzed the response to Cd^2+^ of mutant yeast cells inactivated for non-essential genes encoding key players in these different pathways and whose deletion induced a growth phenotype in the presence of Cd^2+^ (data not shown).

**Figure 2.**
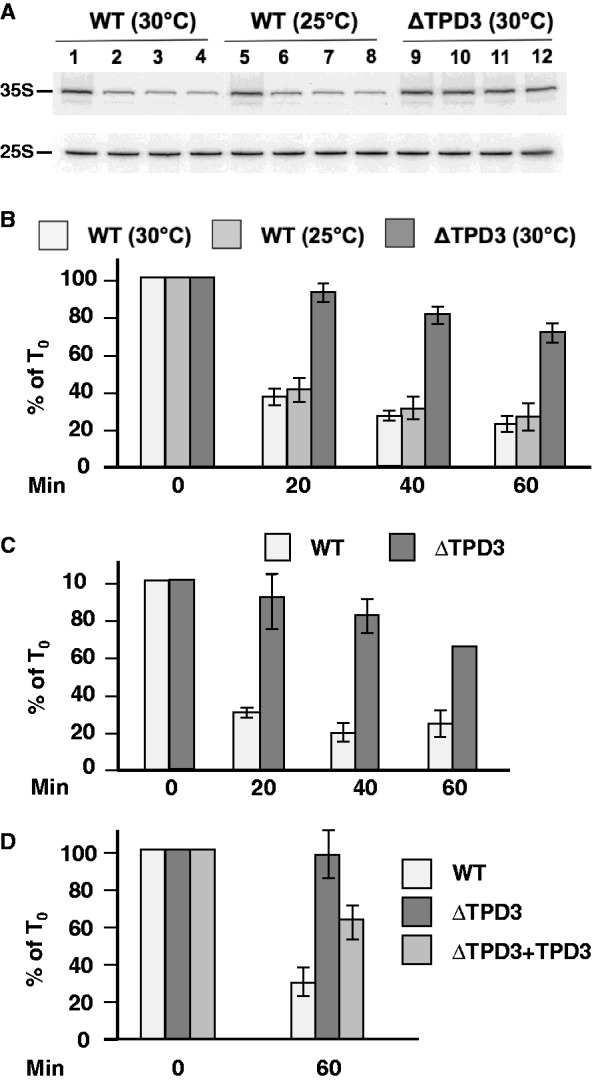
The regulatory subunit TPD3 of the PP2A phosphatase is required to mediate the downregulation of Pol I transcription in response to Cd^2+^. (**A**) WT and ΔTPD3 cells were grown in YPD medium to mid-exponential phase at 25°C or 30°C as indicated, then Cd^2+^ (50 µM) was added (*t* = 0 min) (1, 5, 9) for 20 (2, 6, 10), 40 (3, 7,11) and 60 min (4, 8, 12). For each time of Cd^2+^ treatment, the same number of cells was harvested, total RNAs were extracted and the amount of 35S rRNA was determined by primer extension analysis. (**B**) Quantification of 35S rRNA precursor in three independent primer extension experiments similar to that shown in panel A (100 = amount of 35 S rRNA at *t* = 0). (**C**) Quantification of ChIP analysis of the occupancy of the rDNA unit by Pol I in WT and ΔTPD3 cells treated or not with Cd^2+^ (50 µM) as indicated. (**D**) Quantification of 35S rRNA precursor in WT and ΔTPD3 cells, and in ΔTPD3 cells transformed with a multi-copy plasmid harboring *TPD3* (ΔTPD3+TPD3) treated or not with Cd^2+^ (50 µM) as indicated.

In five of the six deletion mutants (i.e. ΔIRE1, ΔHOG1, ΔSLT2, ΔSNF1 and ΔPDE1), we observed a strong reduction of the level of 35S rRNA during the Cd^2+^ treatment, similar to that observed in isogenic WT control cells (Supplementary Figure S4), strongly suggesting that the corresponding signaling pathways (i.e. ER stress signaling, Pck1, Hog1, Snf1 and cAMP-dependent protein kinase pathways) are not implicated in the Cd^2+^-dependent repression of Pol I transcription. In sharp contrast, cells in which the *TPD3* gene, which encodes the regulatory subunit of PP2A (30), has been deleted behaved differently: the level of the 35S rRNA was significantly less affected in response to Cd^2+^ than was the case with WT cells (Figure 2A, lanes 1–4 and 9–12; Figure 2B, white and dark gray histograms). Indeed, 90% of the 35S RNA signal was still present after 20 min of treatment and 65% after 1 h, instead of 30 and 20%, respectively, for the WT strain (Figure 2B). As expected, analysis by ChIP of Pol I density on the rDNA in WT and ΔTPD3 cells showed that during the course of Cd^2+^ treatment, the decrease of the level of enzyme on the rDNA was significantly attenuated in the ΔTPD3 cells compared with the WT cells, and was, for both strains, comparable with the level of 35S rRNA measured by primer extension (Figure 2C), suggesting that PP2A activity is required for the Cd^2+^-dependent repression of Pol I transcription.

However, in the above experiment, we observed that the doubling time of the ΔTPD3 cells was significantly longer than the doubling time of the WT isogenic control cells (134 min versus 89 min, respectively, at 30°C) in contrast with the ΔIRE1, ΔHOG1, ΔSLT2, ΔSNF1 and ΔPDE1 cells, which all displayed a doubling time identical to that of the WT isogenic control cells (data not shown). Because the transcriptional activity of Pol I, which in turn is directly related to the level of ribosome synthesis, correlates with cell growth (20), we wondered whether the differential Cd^2+^-sensitivity of rRNA synthesis in the ΔTPD3 and WT cells reflected a direct involvement of PP2A in the Cd^2+^-dependent repression of Pol I transcription, or was instead an indirect consequence of the lower metabolic rate of the ΔTPD3 cells. To address this question, we lowered the culture temperature of the WT cells to the point at which their growth rate matched that of the ΔTPD3 mutant cells. We found that the WT strain had the same doubling time at 25°C as the ΔTPD3 mutant cells had at 30°C (132 min versus 134 min). The effect of Cd^2+^ treatment on Pol I transcription was therefore reinvestigated at 30°C for the ΔTPD3 cells and at 25°C for the WT cells. Results of the primer extension analysis (Figure 2A, lanes 1–8; Figure 2B, white and light gray histograms) confirmed that Cd^2+^ treatment led to a similar inhibition of Pol I transcription in the WT cells at 25 and 30°C, demonstrating that the level of Cd^2+^-resistance of Pol I transcription in the ΔTPD3 mutant cells does not result from their reduced growth rate. Taken together, these results point out the central role of PP2A in the inhibition of Pol I transcription in response to Cd^2+^. To confirm the implication of PP2A in the Cd^2+^-dependent repression of Pol I transcription, we first complemented the disruption of *TPD3* by transforming the ΔTPD3 strain with a multicopy plasmid harboring the WT *TPD3* gene under the control of its own promoter (ΔTPD3 + TPD3 strain). As shown in Figure 2D, the 35S RNA level in the transformed cells was significantly more affected by Cd^2+^ treatment than in the isogenic ΔTPD3 cells, although less affected than in control isogenic WT cells. This result confirmed the implication of PP2A in the repression of Pol I transcription in response to Cd^2+^ treatment.

Next, we used another mutant strain defective in PP2A activity. In *S.cerevisiae*, Pph21p, the catalytic subunit of PP2A, is functionally redundant with Pph22p. Double deletion mutant cells (*Δpph21Δpph22*) are viable and do not display any significant growth phenotype, but are synthetic lethal with the deletion of *PPH3*, which encodes another catalytic subunit partially redundant with Pph21p and Pph22p (31). However, the DEY217 mutant cells (*Δpph21Δpph3*, *pph22-172*) are viable, but display a strong temperature-sensitive phenotype and are defective in the catalytic activity of PP2A (22). We therefore compared the effect of Cd^2+^ treatment on Pol I transcription in the thermosensitive DEY217 mutant cells and in WT isogenic cells. Because the growth rate of the DEY217 triple mutant was already affected at 23°C (permissive temperature), we chose not to further perturb the cells by superimposing a temperature stress and therefore performed experiments at this temperature. The 35S rRNA level was monitored by primer extension analysis. As previously observed in the ΔTPD3 strain, the 35S RNA level in the DEY217 triple mutant was only slightly affected by Cd^2+^ treatment, and still represented ∼80% of the initial level after 60 min of Cd^2+^ treatment (Supplementary Figure S5A and B). Accordingly, ChIP analysis indicated that Pol I occupancy of the rDNA unit promoter in the DEY217 triple mutant was only slightly affected, with 70% of the enzyme molecules still present at the promoter after 60 min of Cd^2+^ treatment (Supplementary Figure S5C). Taken together, these results confirm that the repression of Pol I transcription induced by Cd^2+^ is mediated by PP2A.

### Cd^2+^-dependent repression of Pol I transcription targets the stability of the Pol I–Rrn3 complex

**Figure 3.**
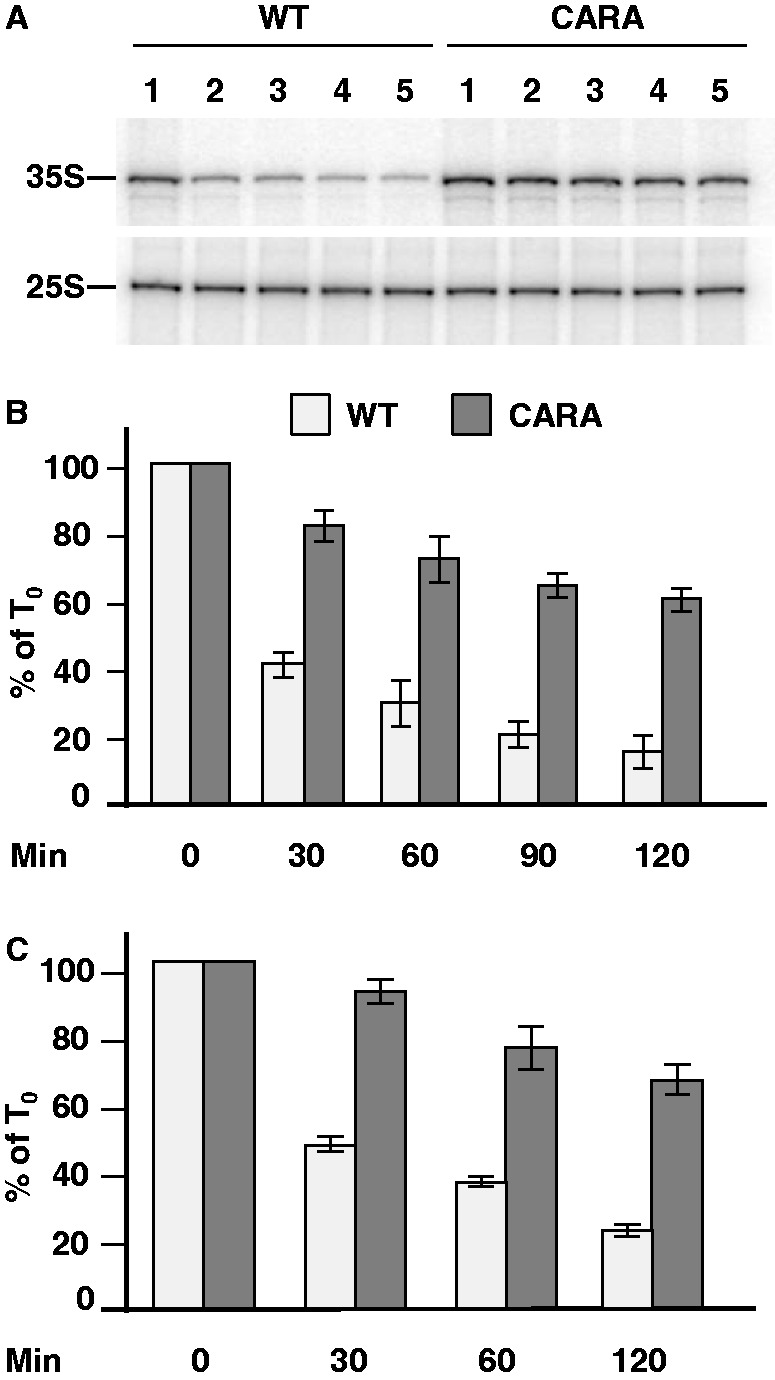
PP2A targets the Pol I–Rrn3 complex in response to Cd^2+^. (**A**) Primer extension analysis of 35S rRNA in WT and in CARA cells. Cells were grown in YPD medium to mid-exponential phase, then Cd^2+^ (50 µM) was added (*t* = 0 min) (1) for 30 (2), 60 (3), 90 (4) and 120 min (5). At each time, the same number of cells was harvested, total RNAs were extracted, and the amount of 35S rRNA was determined by primer extension analysis. (**B**) Quantification of 35S rRNA precursor in four independent primer extension experiments similar to that shown in panel A (100 = amount of 35 S rRNA at *t* = 0). (**C**) ChIP analysis of the occupancy of the 35 S rDNA unit by Pol I. Chromatin extracts were prepared from the same number of WT or CARA cells grown in YPD medium to mid-exponential phase in the presence of Cd^2+^ (50 µM) for various times as indicated. ChIP was performed using anti-A190 antibodies, and quantification was performed by real-time PCR using four pairs of primers hybridizing at the promoter and in three regions along the coding sequence of the rDNA (see ‘Materials and Methods’ section). The occupancy of rDNA by Pol I being nearly identical for each of these four regions, results were averaged and expressed as a percentage of the occupancy seen in absence of Cd^2+^. Three independent ChIP experiments were performed.

The above results indicate that the Cd^2+^-dependent repression of Pol I transcription is mediated by PP2A, which likely affects, directly or indirectly, the initiation step of rDNA transcription. We thus postulated that the molecular target of this regulation within the class I transcriptional machinery may be a class I GTF. In the yeast *S.cerevisiae*, four GTFs are required for Pol I transcription: the multimeric complexes upstream activating factor (UAF) and core factor (CF), which bind onto the bipartite rDNA promoter in association with TATA-binding protein (TBP), and the monomeric transcription factor Rrn3, which interacts with the enzyme to form the Pol I–Rrn3 complex (32,33). This complex is the only initiation-competent form of Pol I (32,34–36) and its formation (or stability) is highly regulated *in vivo*. In mouse, it has been demonstrated that, depending on the stress conditions, different signal transduction pathways mediate the downregulation of Pol I transcription through modifications of the phosphorylation status of TIF-IA (the mouse ortholog of Rrn3), which impairs its interaction with Pol I (37–41). Although in yeast the stability of the Pol I–Rrn3 complex has been shown to be the key element of most, if not all, growth-dependent (23) or rapamycin-dependent (19) repression mechanisms affecting rDNA transcription, it is tempting to speculate that in response to Cd^2+^ treatment, the formation of the Pol I–Rrn3 complex is also hampered. To test this hypothesis, we analyzed the effect of Cd^2+^ treatment on Pol I transcription in mutant cells in which the Pol I–Rrn3 complex is not dissociable. We previously constructed such a strain, named CARA (for Constitutive Association of Rrn3 and A43), in which the genes encoding A43, the Pol I subunit interacting with Rrn3, and Rrn3 are inactivated and complemented by a gene encoding the Rrn3–A43 fusion protein (19). Therefore, WT and CARA cells, which display the same doubling time, were submitted to Cd^2+^ treatment during a time-course analysis, and the *in vivo* level of 35S rRNA was determined by primer extension analysis. In contrast to WT cells, in CARA cells the 35S rRNA level decreased only slightly during Cd^2+^ treatment: after 2 h, it corresponded to 70% of the amount of 35S RNA present in non-treated cells, whereas it dropped to only 18% in WT cells during the same time (Figure 3A and B). ChIP analysis of Pol I present on the rDNA corroborated these results (Figure 3C): occupancy of the rDNA units by Pol I was only slightly affected by Cd^2+^ treatment in CARA cells, whereas it was strongly reduced in WT cells. Again, a robust correlation was found between the level of 35S rRNA and the level of Pol I occupancy of the rDNA during Cd^2+^ treatment in both WT and CARA cells, reinforcing the idea that Cd^2+^-dependent repression of Pol I transcription is mostly exerted at the initiation step of transcription. Because the only difference between the Pol I transcriptional machineries of the WT and CARA strains is the fusion between Rrn3 and the Pol I, these results strongly suggest that, in yeast, the downregulation of Pol I transcription in response to Cd^2+^ is exerted through the PP2A-dependent formation/stability of the Pol I–Rrn3 complex.

### Cd^2+^ ions target the association/dissociation of Rrn3 with Pol I through PP2A

**Figure 4.**
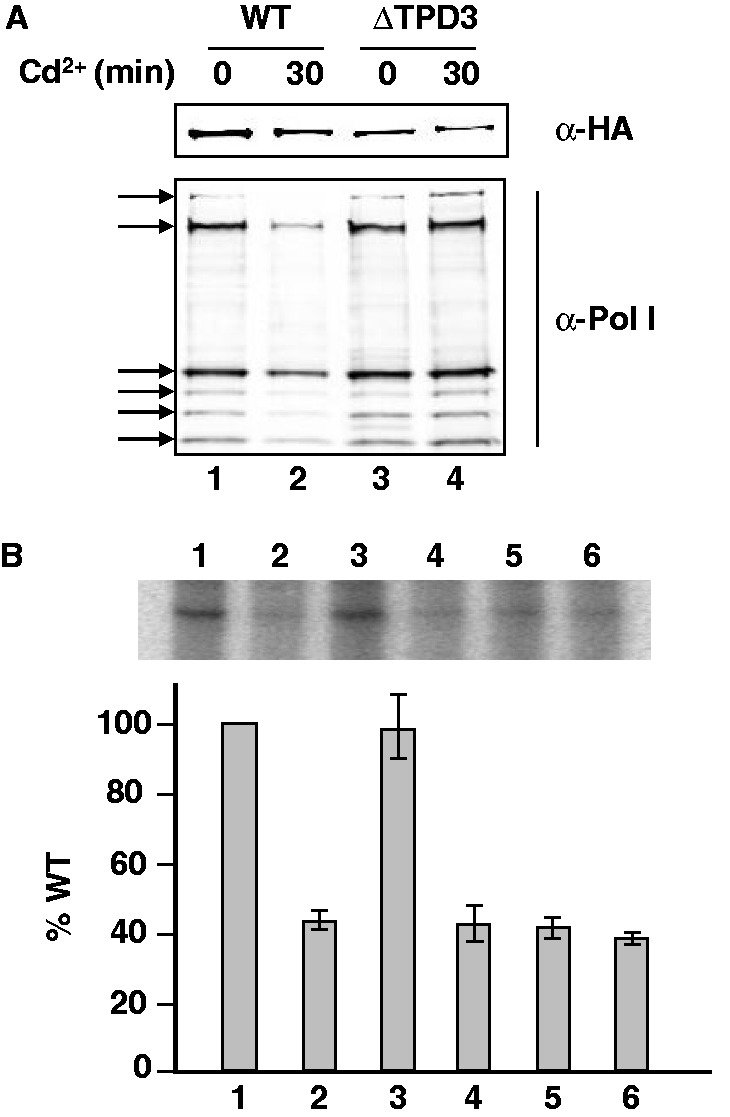
Cd^2+^ ions target the association/dissociation of Rrn3 with Pol I through PP2A. (**A**) Co-immunoprecipitation of Rrn3 and Pol I. WT and ΔTPD3 cells containing a HA-tagged version of Rrn3 were grown in YPD medium to 0.8 OD_600_ at 30°C, then Cd^2+^-treated (50 µM) for 30 min or not. HA-Rrn3 was immunoprecipitated from crude extracts with magnetic beads coated with anti-HA. After washing, bound proteins were eluted and analyzed by western blotting. Membranes were probed with anti-HA and anti-Pol I antibodies for the detection of Rrn3 (upper panel) and Pol I (lower panel), respectively. Immunocomplexes were detected with secondary antibodies conjugated to infrared dyes, scanned and analyzed with the Odyssey imaging system and its associated software (Li-Cor). Positions of Pol I subunits are indicated by arrow. (**B**) Purified Pol I–Rrn3 complex reactivates a partially inactive PA600 extract prepared from Cd^2+^-treated WT cells. PA600 fractions were purified from WT cells (1) or WT cells treated with Cd^2+^ (50 µM) for 30 min (2–6). Partially inactive PA600 from Cd^2+^-treated cells (2) was pre-incubated with purified Pol I–Rrn3 complex (3), rCF (4), rTBP (5) or rUAF (6) before addition of the NTPs to start the *in vitro* transcription. Activity was expressed as the percent of activity of PA600 in extracts from untreated WT cells.

To clearly show that Cd^2+^ targeted the association/dissociation of Rrn3 with Pol I through PP2A, the amount of Pol I–Rrn3 complex was investigated by co-immunoprecipitation experiments in WT and ΔTPD3 cells treated or not with Cd^2+^ for 30 min. Rrn3 was immunoprecipitated with anti-HA antibodies from crude extracts prepared from WT and ΔTPD3 cells expressing a HA-tagged version of Rrn3, and co-immunopurified Pol I was analyzed by western blot with polyclonal antibodies raised against Pol I (26). As shown in the lower panel of Figure 4A, after 30 min of Cd^2+^ treatment, the amount of Pol I co-immunoprecipitated with Rrn3 (i.e. the amount of Pol I–Rrn3 complex) significantly decreased in WT cells (compare lanes 1 and 2), whereas it remained almost constant in ΔTPD3 cells (compare lanes 3 and 4). Because the *in vivo* amount of both Rrn3 and Pol I in WT and ΔTPD3 cells was not significantly modified by a 30-min Cd^2+^ treatment (Supplementary Figure S6), these results indicate that in WT cells, the stability of the Pol I–Rrn3 complex is affected by Cd^2+^ treatment, whereas it remains unchanged in ΔTPD3 cells.

To confirm this result, we next carried out *in vitro* Pol I specific transcription using partially purified extracts (PA600). As expected, PA600 extracts prepared from Cd^2+^-treated WT cells partially lost rDNA transcriptional activity compared with PA600 extracts prepared from untreated cells (Figure 4B, lanes 1 and 2). Remarkably, extracts from Cd^2+^-treated cells retained *in vitro* the same level of activity (i.e. ∼40%) as the *in vivo* level of rDNA transcription estimated by primer extension analysis and ChIP analysis of Pol I on the rDNA promoter (see Figure 1). The partially inactive extract from Cd^2+^-treated WT cells was fully reactivated by adding back purified Pol I–Rrn3 complex (Figure 4B, compare lanes 1 and 3). In contrast, purified recombinant CF, TBP or UAF was not able to reactivate the extract from Cd^2+^-treated cells (Figure 4B, lanes 4–6).

Taken together, these results demonstrate that the Pol I–Rrn3 interaction is the major target of Cd^2+^-dependent repression of rDNA transcription.

### PP2A and metal-dependent repression of Pol I transcription

**Figure 5.**
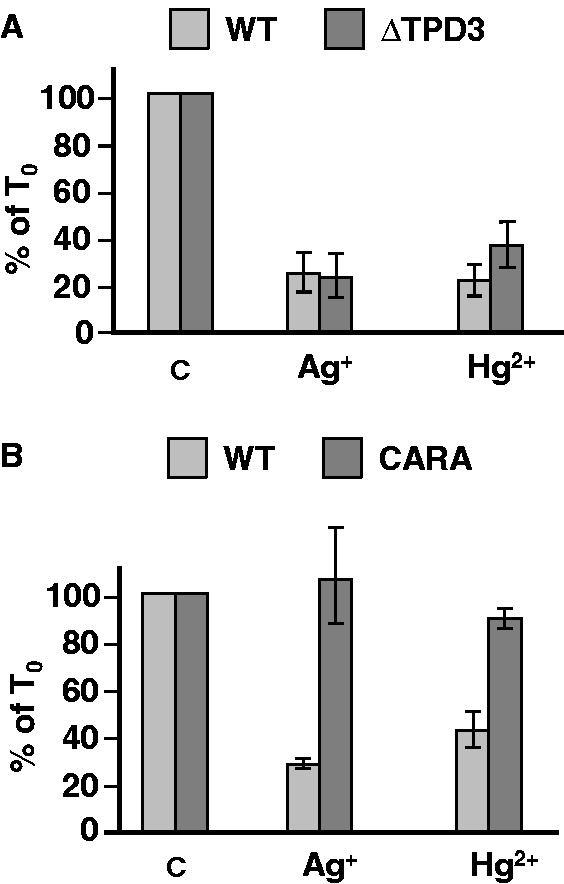
Multiple signal transduction pathways mediating the response to toxic metals target the Pol I–Rrn3 complex. Primer extension analysis of 35S rRNA in ΔTPD3 strain (**A**) or CARA strain (**B**) treated with Ag^+^ (200 µM) or with Hg^2+^ (600 µM). The metal was added (*t* = 0 min) to mid-exponential phase cells grown in YPD medium. The same number of cells was harvested at *t* = 0 and *t* = 60 min, then total RNAs were extracted, and the amount of 35S rRNA was determined by primer extension analysis (100 = amount of 35 S rRNA at *t* = 0).

Finally, we wondered whether repression of Pol I transcription in response to a variety of toxic metals other than Cd^2+^ was, on the one hand, mediated through the stability of the Pol I–Rrn3 complex, and, on the other hand, triggered by PP2A. We therefore analyzed the effect of two other redox-inactive transition metals (Ag^+^ and Hg^2+^) on Pol I transcription in WT and ΔTPD3 strains. Primer-extension analyses after 60 min of treatment showed that Pol I transcription, monitored by the *in vivo* 35S rRNA level, was drastically repressed when WT cells were treated with Ag^+^ (200 µM) or with Hg^2+^ (600 µM) (Figure 5A and B). The level of this repression, ∼80%, is comparable with, although slightly higher than, that observed with Cd^2+^ (50 µM) (compare Figures 2B and 5A and B). Interestingly, the same level of repression was observed in the presence of Ag^+^ or Hg^2+^ when the PP2A function was altered by disruption of the *TPD3* gene (Figure 5A). This result demonstrates that, contrary to what is observed for Cd^2+^, PP2A is not a key player for the repression of Pol I transcription in response to Ag^+^ or to Hg^2+^. Therefore, depending on the nature of the toxic metal, different mechanisms are activated to downregulate Pol I transcription. To determine whether the stability of the Pol I–Rrn3 complex is the molecular target of the Ag^+^- or Hg^2+^-dependent repression mechanisms, WT and CARA cells were treated with these two metals, and the level of 35S RNA was determined by primer extension analysis. Results shown in Figure 5B showed that the level of 35S RNA was not significantly affected in CARA cells treated with Ag^+^ or Hg^2+^, indicating that downregulation of Pol I transcription by these toxic metals does not occur in this strain. Therefore, despite the fact that different toxic metals activate distinct mechanisms to repress Pol I transcription, in all cases tested, the primary target of regulation by these pathways is the formation/dissociation of the Pol I–Rrn3 complex.

## DISCUSSION

Here, we demonstrate that in response to Cd^2+^ treatment, yeast cells rapidly and abruptly shut down Pol I transcription, which represents the main transcriptional activity of the cell. Our data indicate that Cd^2+^ does not affect the Pol I transcriptional machinery by poisoning Pol I and/or a class I GTF, but rather represses 35S rRNA synthesis through a signal transduction pathway. In yeast, low doses of Cd^2+^ were recently shown to cause ER stress that induced the unfolded protein response (UPR) (7). Our results strongly suggest that ER stress and/or UPR are not involved in the Cd^2+^-dependent repression of Pol I transcription. Indeed, deletion of the *IRE1* gene, which encodes a serine/threonine kinase, that is the sensor for an unfolded protein state that transmits the signal across the ER or inner nuclear membrane (42,43), does not interfere with the repression of Pol I activity by Cd^2+^ treatment (Supplementary Figure S4). Instead, we found that the PP2A was necessary for mediating the Cd^2+^-dependent repression of Pol I transcription. PP2A is an essential intracellular serine/threonine phosphatase with broad substrate specificity that can also operate as a phosphotyrosyl phosphatase (44). This phosphatase has repeatedly been shown to play important roles in cytoplasmically localized signal transduction activities. For example, it regulates, directly or indirectly, the Raf-1/MEK/ERK (45), MAP kinase (46), ATM/ATR signaling (47), PP2A-PKCzeta signaling (48), Sp1/PP2A/pRb (49) and Greatwall-PP2A (50) pathways. In addition, and interestingly, an increasing amount of data emphasizes the links existing between PP2A phosphatase and the TOR signaling pathway: Tap42, a regulatory subunit of PP2A, is a downstream effector of the TOR protein kinase, which regulates cell growth in coordination with nutrient and environmental conditions in yeast and mammals; deletion of *TPD3* is colethal with *TCO89*, encoding a subunit of TORC1 (51), and PP2A regulates the TORC2 signaling pathway (52); TOR stimulates growth-promoting association of Tap42 with Pph21/22 and Sit4, whereas Cdc55 and Tpd3 inhibit this association both by direct competition and by dephosphorylation of Tap42 (53). Because (i) we show in this article that the Cd^2+^-dependent repression of Pol I transcription targets the formation/dissociation of the Pol I–Rrn3 complex, (ii) the stability of this complex (Pol I–TIF1A in mouse) is the target element, in the class I transcriptional machinery, for the rapamycin-dependent repression of Pol I transcription in yeast and mammals (19) and (iii) PP2A is a central TOR pathway phosphatase, we wondered whether the PP2A-dependent repression of Pol I transcription during Cd^2+^ treatment was independent of the TOR signaling pathway. To address this question, we compared the extent of rapamycin-dependent repression of Pol I transcription in ΔTPD3 cells with that of WT isogenic control cells. The 35S rRNA level, estimated by primer extension analysis, was similarly affected in both types of cells in response to rapamycin, only 20% of the 35S rRNA signal still being present in both ΔTPD3 and WT cells after 60 min of treatment (Supplementary Figure S7). In other words, rapamycin treatment leads to a similar inhibition of Pol I transcription in ΔTPD3 and WT cells, demonstrating that rapamycin-dependent Pol I transcription repression, in contrast to repression induced by Cd^2+^, does not require the PP2A activity. Finally, a global transcriptomic study showed that Cd^2+^ treatment decreased CK2 and PKA expression in yeast cells (6). These two kinases have been shown to be involved in 35S pre-rRNA synthesis and/or processing (54–56). Accordingly, we cannot formally exclude that CK2 and PKA contribute to the decrease of the level of 35S pre-rRNA during Cd^2+^ treatment. However, our primer extension and ChIP data obtained with the ΔTPD3 and DEY217 mutant strains indicate that the Cd^2+^-dependent repression of rRNA synthesis is predominantly driven by PP2A, at least for treatment times <2 h.

Next, we investigated the molecular mechanisms driving the PP2A-dependent repression of Pol I transcription during Cd^2+^ treatment. The *in vivo* amount of detected 35S pre-rRNA is a balance between synthesis (i.e. Pol I transcription), processing and degradation, and may consequently depend on several different mechanisms. During Cd^2+^ treatment, we observed a strict correlation between the level of 35S pre-rRNA and the density of Pol I on the rDNA promoter for all yeast strains studied (i.e. WT, ΔTPD3 and CARA: see Figures 1–3, respectively). This observation suggests that the *in vivo* level of 35S rRNA during Cd^2+^ treatment is mostly driven by the transcriptional activity of Pol I, and that possible secondary effects of this metal on processing and/or degradation of 35S rRNA do not have any significant influence on the level of rRNA precursor. This conclusion is confirmed by genetic, biochemical and functional approaches, which highlight the critical role of the Pol I–Rrn3 complex in the Cd^2+^-dependent repression of Pol I transcription. This complex is the only initiation-competent form of Pol I (32,34–36), confirming that Cd^2+^-dependent repression of Pol I transcription is regulated at the initiation level. The involvement of PP2A in these mechanisms suggests that Cd^2+^ may induce or change, directly or indirectly, the phosphorylation pattern of the Pol I–Rrn3 complex. In yeast, both Rrn3 and Pol I are phosphorylated *in vivo* (57–59). Association of Pol I with Rrn3 is paralleled by a change in the Pol I phosphorylation pattern, and dephosphorylation of Pol I *in vitro* reduced the initiation activity of yeast Pol I. It also resulted in destabilization of the preformed Pol I–Rrn3 complex, suggesting that the phosphorylation level of Pol I may modulate the Pol I–Rrn3 interaction (59). In mammals, TIF-IA [the mouse ortholog of yeast Rrn3 (36)] is phosphorylated at multiple sites, and it has been shown that both the phosphorylation pattern and the activity of TIF-IA (i.e. its ability to interact with Pol I) are altered in response to environmental changes (39,40), but the phosphorylation status of Pol I and its possible implication in the stability of the Pol I–TIF-IA complex were not investigated in those studies. Taken together, these data suggest that stability of the Pol I–Rrn3 complex may be regulated by modifying the phosphorylation status of Pol I and/or Rrn3. Characterization of the residues involved is still an open question, but the absence of a consensus sequence for PP2A-dependent dephosphorylation, the number of potential target proteins (i.e. Rrn3 and the 14 subunits of Pol I) and the fact that only minor proportions of Pol I molecules and of Rrn3 molecules (≤1%) are engaged within the Pol I–Rrn3 complex make it difficult to address.

The Pol I–Rrn3 complex is conserved throughout evolution because in mammals the initiation-competent form of Pol I is also recruited onto the promoter as a complex with hRrn3/TIF-IA, and because its key role in the regulation of rRNA synthesis is also conserved. Indeed, in mammals, nutrient starvation, density arrest, protein synthesis inhibitors and growth-factor–dependent activation of ribosome biogenesis all lead to Pol I transcription repression through cellular signaling cascades that directly target TIF-IA, whose reversible phosphorylation regulates its association with Pol I and hence transcription initiation complex formation [for review, see (34)]. JNK-mediated phosphorylation of TIF-IA also plays a key role in oxidative- and ribotoxic stress-dependent regulation of rRNA synthesis (40). Regulation of Pol I transcription in mammals is not, however, controlled only by hRrn3/TIF-IA–Pol I complex stability. Indeed, reversible acetylation and phosphorylation of basal components of the Pol I transcription machinery other than hRrn3/TIF-IA (i.e. UBF and TIF-1B/SL1) may be an effective means of regulating rDNA transcription [for review, see (34)].

In yeast, specific transcription initiation by Pol I requires four general initiation factors: the UAF, the TBP, the CF and Rrn3. Assembled onto the 35S rDNA promoter, UAF, TBP and CF form a pre-initiation complex that recruits the Pol I–Rrn3 complex, the only form of Pol I that is competent for transcription initiation. Within the class I machinery in yeast cells, apart from the particular case in which UAF plays an essential role in silencing Pol II transcription of rDNA (60), the only target for the molecular mechanisms controlling the level of 35S rRNA synthesis during nutrient starvation (6) or rapamycin treatment (19) to be identified thus far is the control of the stability of the Pol I–Rrn3 complex. Our study demonstrates that metals repressing Pol I transcription likewise target the formation/dissociation of the Pol I–Rrn3 complex, thereby highlighting the central and unique role of this complex in regulation of Pol I transcription in situations unrelated to growth-dependent repression.

## SUPPLEMENTARY DATA

Supplementary Data are available at NAR Online: Supplementary Figures 1–7.

## FUNDING

French National Agency for Research [ANR 08 PCVI 0036]; Fellowship from the French-Chinese Foundation for Science and its Applications (to L.Z.). Funding for open access charge: French National Agency for Research [ANR 08 PCVI 0036] and French Alternative Energies and Atomic Energy Commission.

*Conflict of interest statement.* None declared.

## Supplementary Material

Supplementary Data
