# Supplementary material for: Repression of class I transcription by cadmium is mediated by the protein phosphatase 2A
Source: Nucleic Acids Res. 2013 May 2;41(12):6087–97. doi: 10.1093/nar/gkt335 (PMC3695495; doi:10.1093/nar/gkt335)
Supplement: Supplementary Data [file supp_41_12_6087__index.html]

Repression of class I transcription by cadmium is mediated by the protein phosphatase 2A — Repression of class I transcription by cadmium is mediated by the protein phosphatase 2A — Supplementary Data 

# Repression of class I transcription by cadmium is mediated by the protein phosphatase 2A

## Supplementary Data

files

**Files in this Data Supplement:**

- Supplementary Data - pdf file
