# Supplementary material for: Repression of class I transcription by cadmium is mediated by the protein phosphatase 2A
Source: Nucleic Acids Res. 2013 May 2;41(12):6087–97. doi: 10.1093/nar/gkt335 (PMC3695495; doi:10.1093/nar/gkt335)

Supplementary Figure Legends

Supplementary Figure 1: Exposure of yeast cells to low concentrations of Cd^{2+} led to modest cellular mortality. WT and CARA cells were exposed to 50 µM or 100 µM of Cd^{2+} for 30 min and 60 min, then a fixed number of cells was plated on YPD medium and incubated at 30°C. Number of live cells was determined by counting. Results were expressed as the percentage of live cells seen in absence of Cd^{2+}-treatment.

Supplementary Figure 2: Cd^{2+}-treatment inhibited Pol I occupancy of the rDNA unit. A) Schematic representation of a rDNA unit from *Saccharomyces cerevisiae*. The position of the different probes, used for ChIP analysis, over the promoter (P) and the unit (E1 to E3) is indicated. The arrow shows the transcription start site. B) ChIP analysis of the occupancy of the rDNA unit by Pol I. Cells were grown in YPD medium to mid-exponential phase and Cd^{2+} (50 µM) was added for 60 min. Chromatin extracts were then prepared from the same numbers of cells at the indicated times. ChIP was performed using polyclonal antibodies raised against A190, the largest Pol I subunit, and quantification was performed by real-time PCR. The occupancy of the rDNA unit by Pol I after Cd^{2+}-treatment is represented as the mean percentage of the occupancy seen in the absence of Cd^{2+} in 4 independent experiments.

Supplementary Figure 3: Cd^{2+} does not inhibit Pol I specific transcription *in vitro*. A partially purified yeast extract fraction (PA600) containing the components required for the specific *in vitro* transcription of a rDNA unit was preincubated in the presence of various concentrations of Cd^{2+} (as indicated) together with a plasmid harboring a mini-rDNA unit. Transcription was started by addition of the NTPs.

Supplementary Figure 4: ER, Hog1, Pkc1, Snf1, and cAMP-dependent PK signaling pathways are not involved in the repression of Pol I transcription by Cd^{2+}. Primer extension analysis of 35S rRNA in wild-type cells (WT) and in deletion mutants of genes belonging to distinct signal transduction pathways. Cells were treated with Cd^{2+} (50 µM) during 0 min (1), 20 min (2), 40 min (3), and 60 min (4). At these times, total RNAs were extracted from the same number of cells and analyzed.

Supplementary Figure 5: Down-regulation of Pol I transcription in response to Cd^{2+} requires PP2A. A) Determination of the amount of 35S rRNA by primer extension analysis in wild-type (WT) and in DEY217 cells. DEY217 cells are defective in PP2A catalytic activity as a result of deletions of the *PPH21* and *PPH3* genes in combination with the thermosensitive allele pph22-172. Cells were grown in YPD medium to mid-exponential phase, then Cd^{2+} (50 µM) was added (t = 0 min) (1) for 30 min (2), 60 min (3), and 90 min (4). At these times, total RNAs were extracted from the same number of cells and analyzed. (B) Quantification of 35S rRNA precursor in 3 independent primer extension experiments similar to that shown in panel A (100 = amount of 35 S rRNA at t=0). (C) ChIP analysis of the occupancy of the rDNA unit by Pol I. Cells were grown in YPD medium to mid-exponential phase and Cd^{2+} (50 µM) was added for 60 min. Chromatin extracts were then prepared from the same

number of cells. ChIP was performed using polyclonal antibodies raised against A190, the largest Pol I subunit, and quantification was performed by real-time PCR. The occupancy of the rDNA unit by Pol I after Cd^{2+} treatment is represented as the percentage of the occupancy in the absence of Cd^{2+} in 4 independent experiments.

Supplementary Figure 6: Cd^{2+}-treatment does not modify the *in vivo* amount of Rrn3 and Pol I. WT and ∆TPD3 cells containing a HA-tagged version of Rrn3 were grown in YPD medium to 0.8 OD_{600} at 30°C, and Cd^{2+}-treated (50 µM for 30 min) or not before disruption in an Eaton press. Proteins of crude extracts (40 µg) were separated by gel electrophoresis in denaturing conditions and blotted onto a nitrocellulose membrane. A) Red Ponceau staining of the membrane. B) Western-blot of the membrane with anti-HA monoclonal antibodies and rabbit anti-Pol I polyclonal antibodies as indicated for the detection of Rrn3 and Pol I respectively. Position of the A135, A49, A43, AC40 and A34.5 Pol I subunits is indicated. Asterisks correspond to the main contaminants recognized by the anti-Pol I polyclonal antibodies.

Supplementary Figure 7: Rapamycin-dependent Pol I transcription repression does not require PP2A activity. Primer extension analysis of 35S rRNA in WT and in ΔTPD3 cells in response to rapamycin added at t=0 to mid-exponential phase cells grown in YPD medium. The same number of cells was harvested at t=0 and t=60 min, then total RNAs were extracted, and the amount of 35S RNA was determined by primer-extension analysis (100= amount of 35S RNA at t=0). The occupancy of the rDNA unit by Pol I after Cd^{2+}-treatment is presented as percentage of the occupancy seen in the absence of Cd^{2+}.

Supplementary Figure 1

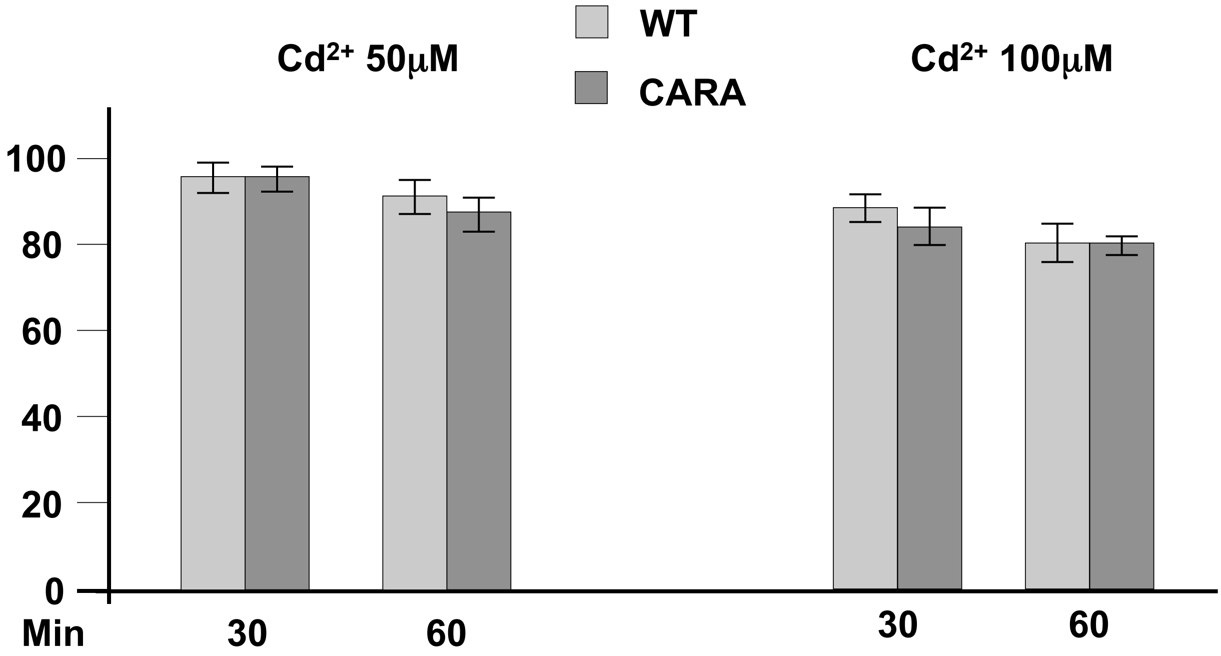

Supplementary Figure 2

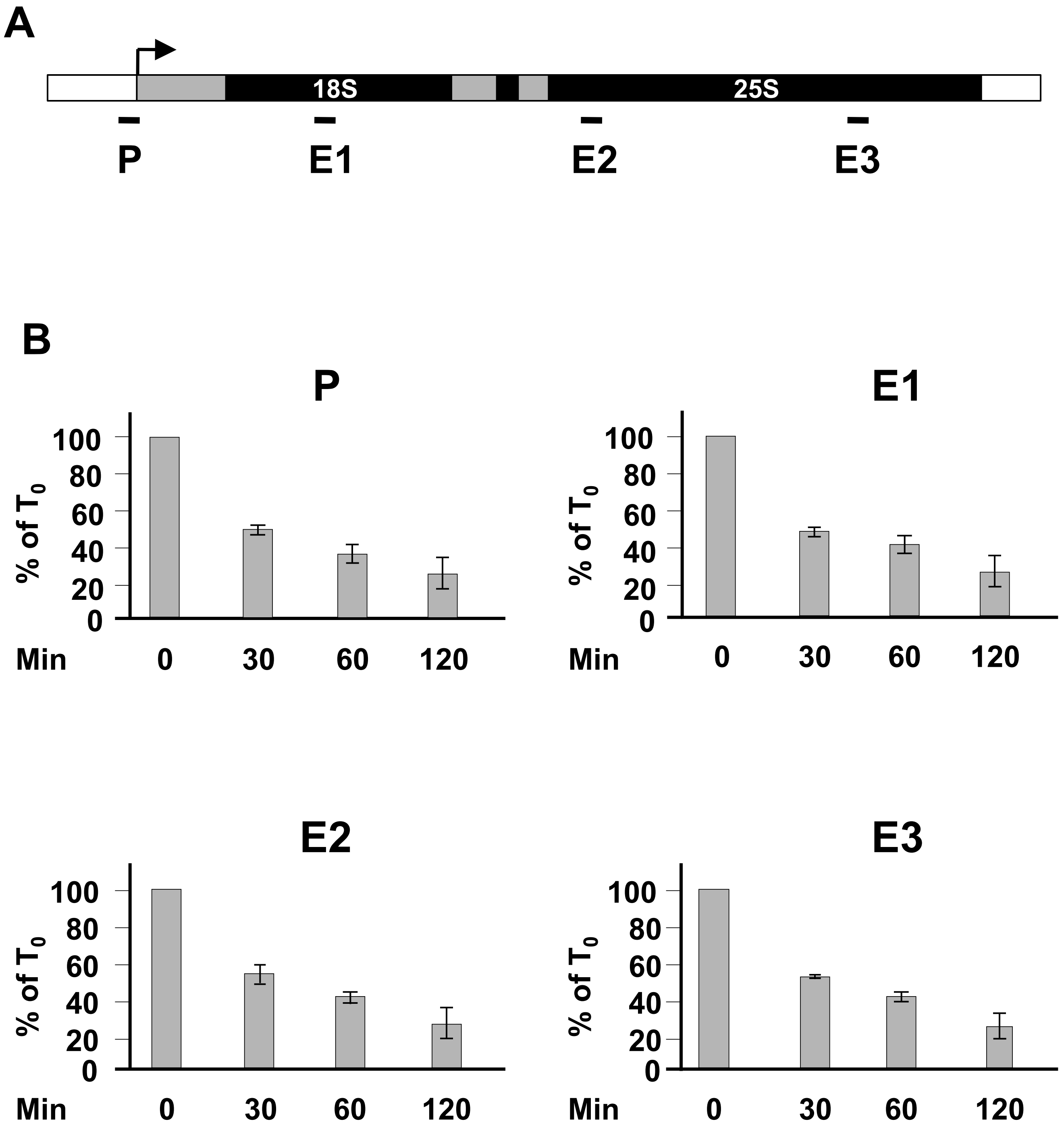

Supplementary Figure 3

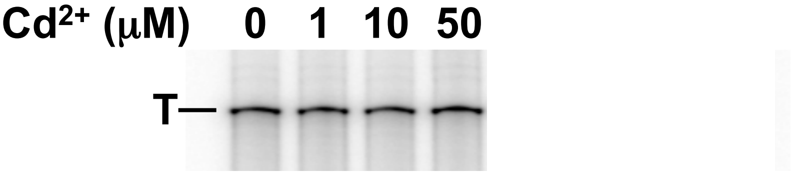

Supplementary Figure 4

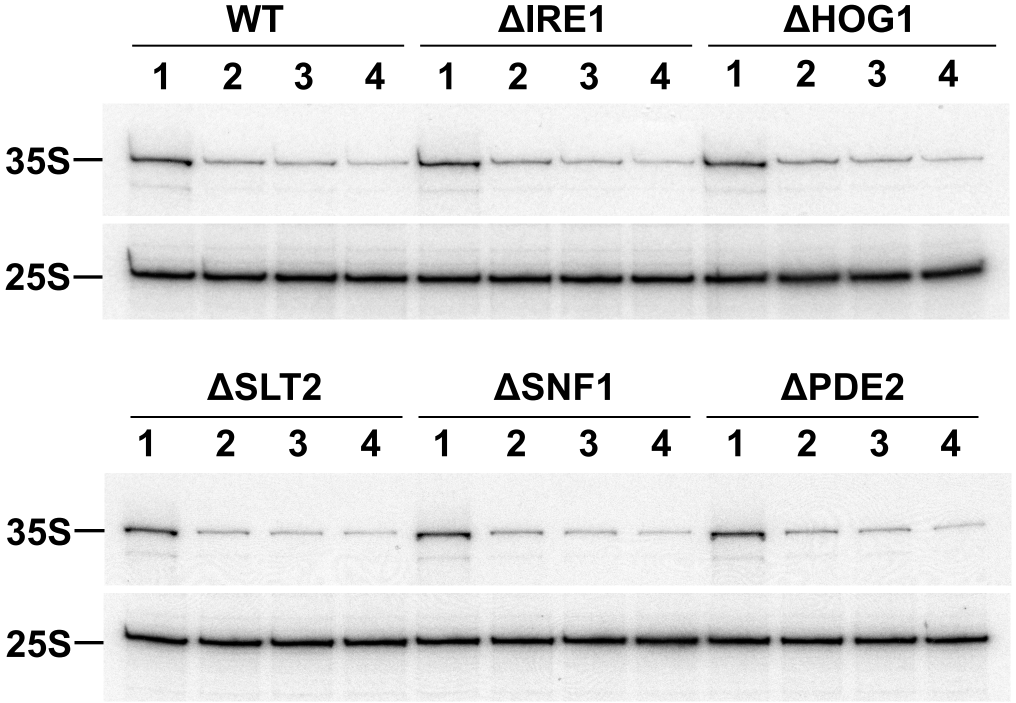

Supplementary Figure 5

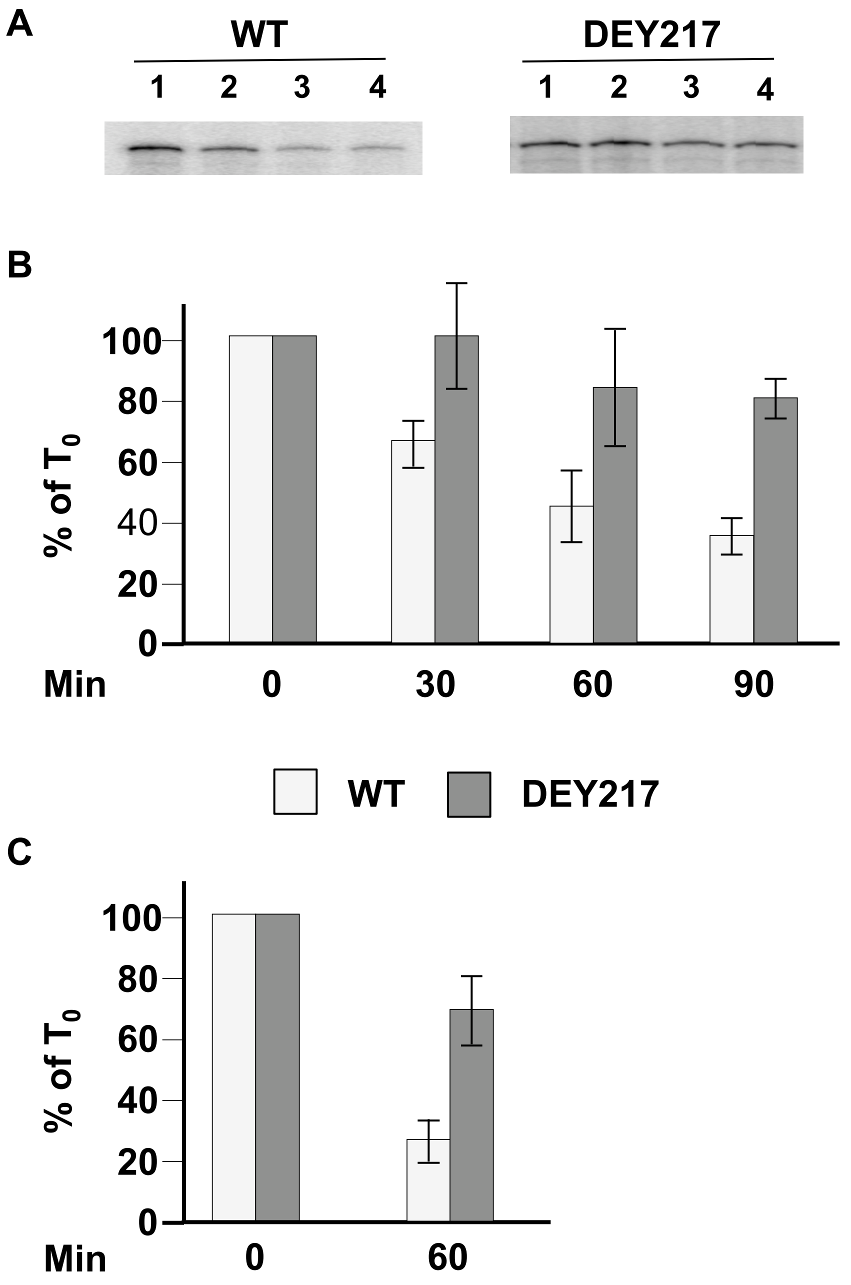

Supplementary Figure 6

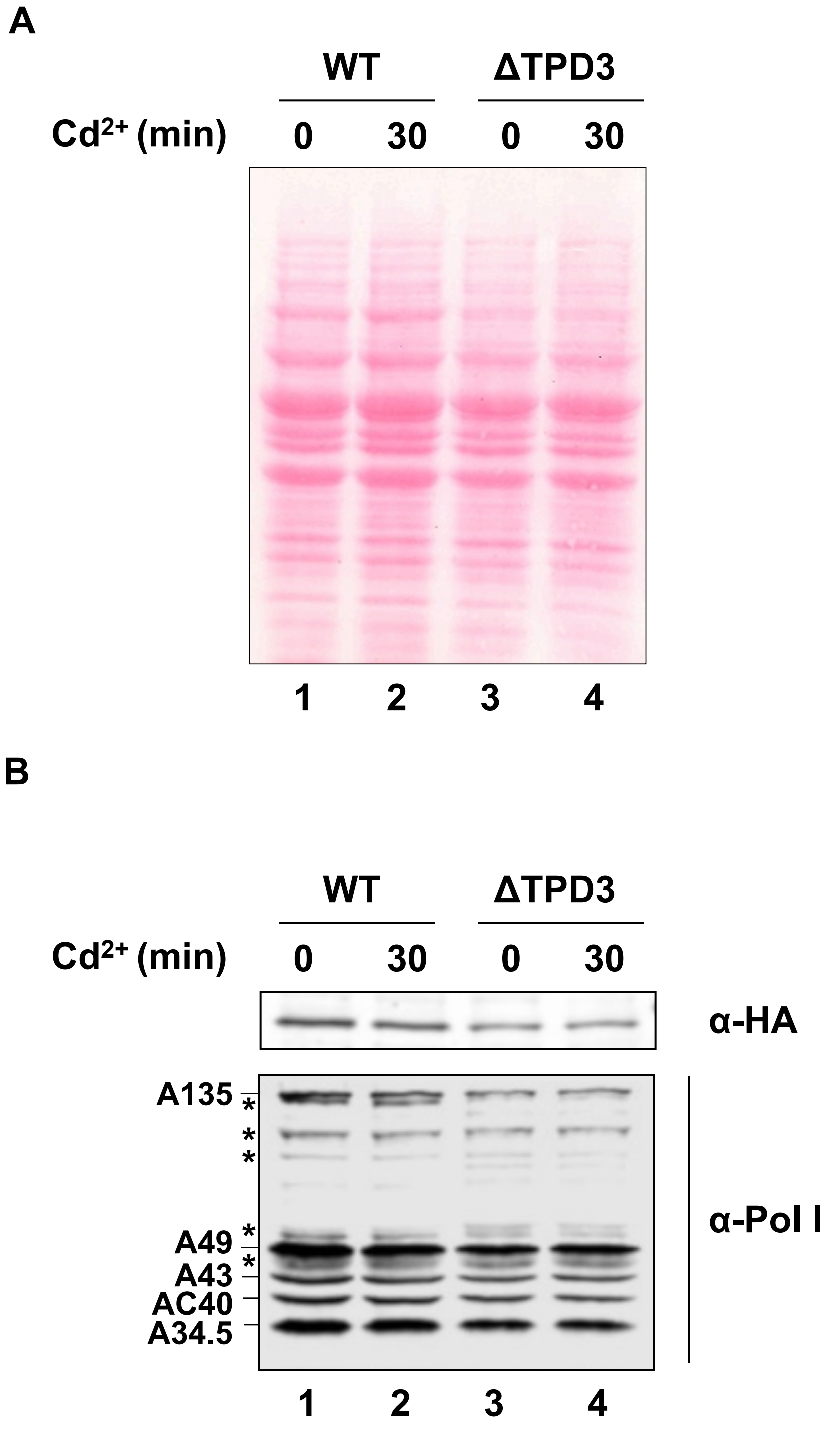

Supplementary Figure 7

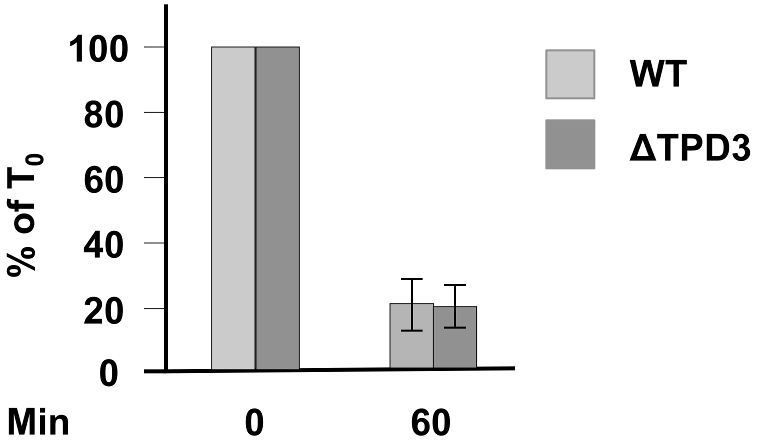

Supplement: Supplementary Data [file supp_gkt335_nar-00768-v-2013-File003.pdf]
